# Process optimization for mass production of 2,3-butanediol by *Bacillus subtilis* CS13

**DOI:** 10.1186/s13068-020-01859-w

**Published:** 2021-01-08

**Authors:** Dexin Wang, Baek-Rock Oh, Sungbeom Lee, Dae-Hyuk Kim, Min-Ho Joe

**Affiliations:** 1 Radiation Utilization and Facilities Management Division, Korea Atomic Energy Research Institute, 29 Geumgu-gil, Jeongeup, 56212 Republic of Korea; 2 Department of Bioactive Material Sciences, Institute for Molecular Biology and Genetics, Center for Fungal Pathogenesis, Jeonbuk National University, Jeonju, 54896 Republic of Korea; 3 Microbial Biotechnology Research Center, Jeonbuk Branch Institute, Korea Research Institute of Bioscience and Biotechnology (KRIBB), Jeongeup, 56212 Republic of Korea; 4 Radiation Research Division, Korea Atomic Energy Research Institute, 29 Geumgu-gil, Jeongeup, 56212 Republic of Korea; 5 Department of Radiation Science and Technology, University of Science and Technology, Daejeon, 34113 Republic of Korea

**Keywords:** 2,3-butanediol, *B. subtilis* CS13, Response surface methodology, Fermentation

## Abstract

**Background:**

*Bacillus subtilis* CS13 was previously isolated for 2,3-butanediol (2,3-BD) and poly-γ-glutamic acid (γ-PGA) co-production. When culturing this strain without L-glutamic acid in the medium, 2,3-BD is the main metabolic product. 2,3-BD is an important substance and fuel with applications in the chemical, food, and pharmaceutical industries. However, the yield and productivity for the *B. subtilis* strain should be improved for more efficient production of 2,3-BD.

**Results:**

The medium composition, which contained 281.1 g/L sucrose, 21.9 g/L ammonium citrate, and 3.6 g/L MgSO_4_·7H_2_O, was optimized by response surface methodology for 2,3-BD production using *B. subtilis* CS13. The maximum amount of 2,3-BD (125.5 ± 3.1 g/L) was obtained from the optimized medium after 96 h. The highest concentration and productivity of 2,3-BD were achieved simultaneously at an agitation speed of 500 rpm and aeration rate of 2 L/min in the batch cultures. A total of 132.4 ± 4.4 g/L 2,3-BD was obtained with a productivity of 2.45 ± 0.08 g/L/h and yield of 0.45 g_2,3-BD_/g_sucrose_ by fed-batch fermentation. The meso-2,3-BD/2,3-BD ratio of the 2,3-BD produced by *B. subtilis* CS13 was 92.1%. Furthermore, 89.6 ± 2.8 g/L 2,3-BD with a productivity of 2.13 ± 0.07 g/L/h and yield of 0.42 g_2,3-BD_/g_sugar_ was achieved using molasses as a carbon source.

**Conclusions:**

The production of 2,3-BD by *B. subtilis* CS13 showed a higher concentration, productivity, and yield compared to the reported generally recognized as safe 2,3-BD producers. These results suggest that *B. subtilis* CS13 is a promising strain for industrial-scale production of 2,3-BD.

## Background

In the past, many bulk chemicals could only be produced by petroleum refining or other chemical processes. Now, however, the production of bulk chemicals by microbial fermentation has been extensively studied. 2,3-butanediol (2,3-BD) production by microbial fermentation is one example. 2,3-BD is mainly used in the food, pharmaceutical, polymers, and cosmetics industries [1,2]. Such as 2,3-BD can be easily converted to 1,3-propanediol (1,3-PD), which can then be used as a monomer and synthesis polytrimethylene terephthalate [3]. Due to its low freezing point, 2,3-BD can be used as an antifreeze agent [4]. Additionally, 2,3-BD is an excellent fuel with a heat value of 27,198 J/g [1]. At present, 2,3-BD is primarily produced by microbial fermentation using renewable energy sources as raw materials, the greatest advantage of which is their environmentally friendly nature. The bacterial species that produce 2,3-BD include *Klebsiella*, *Bacillus*, *Pseudomonas*, and *Serratia* [5–8]. Thus far, *K. pneumoniae* and *K. oxytoca* are considered to be the most promising microorganisms for the production of 2,3-BD due to their high yield and productivity. However, *Klebsiella* spp. are pathogenic microorganisms, which limit their potential for use in the food and pharmaceutical industries. Therefore, the use of non-pathogenic bacteria for industrial production of 2,3-BD is of great significance.

*Bacillus subtilis*, a generally recognized as safe strain, offers numerous benefits in terms of safety. In *B. subtilis*, carbon sources are hydrolyzed and synthesized into pyruvate by glycolysis. Acetoin as a precursor of 2,3-BD is catalyzed by acetolactate synthase and acetolactate decarboxylase, then reduced to 2,3-BD by 2,3-BD dehydrogenase (BDH). *B. subtilis* can produce mixtures of meso-2,3-BD (*R,S*-2,3-BD) and D-2,3-BD (*2R,3R*-2,3-BD) with different ratios according to fermentation conditions. Thus, some research has focused on the production of chiral pure meso-2,3-BD and chiral pure D-2,3-BD using metabolic engineering methods [9, 10]. The yield of 2,3-BD is a determining factor for *B. subtilis* use in industrial applications. The previous study found that oxygen supply favors the high-level production of 2,3-BD [11]. Besides, the type of substrate and concentration, nitrogen source, and inorganic salts also significantly affect the 2,3-BD formation [11–14]. Recently, genetic engineering methods have been widely used to reduce the synthesis of byproducts and increase 2,3-BD production [15, 16]. Furthermore, isolation of high 2,3-BD-producing strains and optimization of fermentation medium and culture conditions are indispensable methods [14, 17].

*B. subtilis* CS13 was previously isolated in our laboratory for the co-production of 2,3-BD and poly-γ-glutamic acid (γ-PGA) [18]. In this study, the fermentation medium for 2,3-BD production was optimized using response surface methodology (RSM). Batch cultures were employed to investigate the best fermentation conditions. Moreover, fed-batch fermentation was carried out to further increase 2,3-BD concentrations using sucrose and untreated molasses as the respective sole carbon sources. To our knowledge, *B. subtilis* CS13 demonstrated a higher titer, yield, and productivity of 2,3-BD compared to existing reports.

## Methods

### Microorganisms and medium

*B. subtilis* CS13 was used for 2,3-BD fermentation. The strain has been deposited into the Korean Collection for Type Cultures (KCTC) with the accession number KCTC 14094 BP.

The basal medium contained 10 g/L tryptone, 5 g/L yeast extract, 10 g/L NaCl, and 20 g/L sucrose. The fermentation medium (pH 6.5) was composed of 250–350 g/L sucrose, 10–30 g/L ammonium citrate, 1 g/L KH_2_PO_4_, 1–5 g/L MgSO_4_·7H_2_O, 0.04 g/L FeCl_3_·6H_2_O, 0.15 g/L CaCl_2_·2H_2_O, 0.12 g/L MnCl_2_·4H_2_O, and 0.5 g/L NaCl.

### Culture conditions

A loopful of *B. subtilis* CS13 from a basal medium agar plate was transferred into a 50-mL tube containing 15-mL fresh basal medium and grown at 37 ℃ with shaking at 200 rpm for 24 h. The pre-culture was then inoculated (1% v/v) into 250-mL flasks containing 50-mL fermentation medium (initial pH 6.5) according to the RSM design. After culturing for 24 h at 37 ℃ with shaking at 200 rpm, 0.5 g sterilized CaCO_3_ was added to each flask to neutralize the acids and maintain a neutral pH.

For bioreactor fermentation, the pre-culture was inoculated (1% v/v) into 500-mL flasks containing 100-mL basal medium, cultured for 12 h, and then transferred to a 3-L fermenter (FMT-ST-D03; Bio System Engineering & Machine Company, Cheongju, Korea) containing 0.9-L optimized fermentation medium with an initial 100 g/L sucrose. The agitation speed and aeration rate were maintained at 400–600 rpm and 1–2 L/min, respectively. For fed-batch culture, the agitation speed and aeration rate were maintained at 500 rpm and 2 L/min, respectively. Bottles of sucrose were added when the total sugar concentration dropped below 20 g/L. The pH was automatically controlled at 6.5 ± 0.1 by adding 2 M NaOH. An initial 100 g untreated molasses, which was purchased from Byeoli Science Co., Ltd. (Jeonju, South Korea) and contained 31.56% (w/w) sucrose, 7.84% (w/w) glucose, and 8.58% (w/w) fructose, was added to the fermenter instead of sucrose. Approximately 150 g molasses was added to the bioreactor when the total sugar concentration fell under 20 g/L.

### RSM experimental design

Previous research has found that sucrose, ammonium citrate, and MgSO_4_·7H_2_O exert significant effects on 2,3-BD production [18]. In this study, the face-centered central composite design (FCCD) was employed to optimize the three most significant variables for further enhancing 2,3-BD production. The independent variables and their coded levels were studied at three different levels (−1, 0, and + 1), including the real value of each level, and a set of 20 experiments was carried out as shown in Table 1. A one-way analysis of variance (ANOVA) and response surfaces were carried out using Design Expert 10.0.6 software (Stat-Ease Corporation, Minneapolis, MN, USA) to investigate the effects of various factors on 2,3-BD production. The yield of 2,3-BD was fitted with the following second-degree polynomial equation:

$$ {\text{Y}} = \beta_{0} + \sum {\beta_{i} X_{i} + } \sum {\beta_{ij} X_{i} X_{j} + } \sum {\beta_{ii} X_{i}^{2} } , $$

**Table 1:** Coded levels and real values of the three variables and responses in the FCCD experiment

Run	Sucrose (g/L)	Ammonium citrate (g/L)	MgSO_4_·7H_2_O (g/L)	2,3-BD (g/L)
1	0 (300)	0 (20)	0 (3)	124.66 ± 4.06
2	1 (350)	− 1 (10)	− 1 (1)	66.78 ± 2.03
3	− 1 (250)	1 (30)	− 1 (1)	105.63 ± 3.42
4	− 1 (250)	− 1 (10)	− 1 (1)	93.10 ± 2.98
5	0 (300)	0 (20)	0 (3)	118.94 ± 3.36
6	0 (300)	1 (30)	0 (3)	115.10 ± 3.12
7	0 (300)	0 (20)	1 (5)	117.61 ± 3.24
8	0 (300)	0(20)	0 (3)	121.03 ± 3.95
9	1 (350)	− 1 (10)	1 (5)	78.36 ± 2.76
10	0 (300)	0 (20)	0 (3)	124.81 ± 4.01
11	0 (300)	− 1(10)	0 (3)	105.99 ± 3.13
12	1(350)	0 20)	0 (3)	95.44 ± 2.99
13	0 (300)	0 (20)	0 (3)	121.68 ± 4.03
14	0 (300)	0 (20)	− 1 (1)	112.10 ± 3.53
15	− 1 (250)	− 1(10)	1 (5)	107.05 ± 3.22
16	− 1 (250)	1 (30)	1 (5)	108.95 ± 3.22
17	1 (350)	1 (30)	1 (5)	94.41 ± 2.87
18	0 (300)	0 (20)	0 (3)	122.08 ± 4.03
19	− 1 (250)	0 (20)	0 (3)	116.76 ± 3.23
20	1 (350)	1 (30)	− 1 (1)	82.99 ± 2.66

where Y is the predicted 2,3-BD concentration (response), *β*_*0*_ is the intercept term, *β*_*i*_ is the linear coefficient, *β*_*ij*_ is the quadratic coefficient, *β*_*ii*_ is the squared term, and *X*_*i*_ and *X*_*j*_ are independent variables.

### Analysis methods

The absorbance of the fermentation broth was determined at 600 nm using a UV–Vis spectrophotometer (Libra S70PC; Biochrom Ltd., Cambridge, England). The cell biomass was calculated using a calibration curve between the optical density at 600 nm and dry cell weight [19].

The concentrations of 2,3-BD and acetoin were determined using an Agilent 1100 high-performance liquid chromatography (HPLC) system equipped with an Aminex HPX-87H column (300 × 7.8 mm; Bio-Rad Laboratories, Hercules, CA, USA) and a refractive index detector. The concentrations of sucrose, glucose, and fructose in the broth were measured using the Aminex HPX-87P column (300 × 7.8 mm; Bio-Rad). The mobile phase consisted of 5 mM H_2_SO_4_ and HPLC-grade water with a flow rate of 0.6 mL/min, and the column temperature was controlled at 65 ℃.

## Results and discussion

### RSM design for 2,3-BD production

The significant independent variables (sucrose, ammonium citrate, and MgSO_4_·7H_2_O) for 2,3-BD production were optimized using the FCCD, and the yields of 2,3-BD as the response are listed in Table 1. A wide range of 2,3-BD titers from 83.0 to 124.8 g/L were observed across the 20 experiments. In this model, a *P*-value < 0.05 was used to indicate significant variables. The results showed that sucrose (*X*_1_), ammonium citrate (*X*_2_), MgSO_4_·7H_2_O (*X*_3_), their squared terms (*X*_1_^2^, *X*_2_^2^, and *X*_3_^2^), and the quadratic terms of sucrose and ammonium citrate (*X*_1_*X*_2_) had significant effects on 2,3-BD production (*P* < 0.05) (Table 2). The second-order polynomial equation for calculating 2,3-BD production (Y) using coded variables was as follows:

$$ {\text{ Y}} = 121.46 - 11.35 \, X_{1} + 5.58 \, X_{2} + 4.58 \, X_{3} + 2.23 \, X_{1} X_{2} - 14.26X_{1}^{2} - 9.82X_{2}^{2} - 5.51X_{3}^{2}.$$

**Table 2:** Statistical analysis of the FCCD for 2,3-BD production by *B. subtilis* CS13

Factors	Mean square	Coefficient estimate	Standard error	*F* value	*P* value
Model	572.55	121.46	0.86	92.13	< 0.0001
*X*_1_: Sucrose	1288.45	− 11.35	0.79	207.34	< 0.0001
*X*_2_: Ammonium citrate	311.36	5.58	0.79	50.10	< 0.0001
*X*_3_: MgSO_4_·7H_2_O	209.58	4.58	0.79	33.73	0.0002
*X*_1_*X*_2_	39.74	2.23	0.88	6.39	0.0299
*X*_1_*X*_3_	4.10	0.72	0.88	0.66	0.4353
*X*_2_*X*_3_	14.55	− 1.35	0.88	2.34	0.1569
*X*_1_^2^	559.24	− 14.26	1.50	89.99	< 0.0001
*X*_2_^2^	264.94	− 9.82	1.50	42.63	< 0.0001
*X*_3_^2^	83.35	− 5.51	1.50	13.41	0.0044
Lack of Fit	7.40			1.47	0.3410
*R*^2^ = 0.9881	*R*^2^_(adj)_ = 0.9774		*R*^2^_(Pred)_ = 0.9054		

The coefficient of determination (*R*^2^ = 0.9881) indicated that 98.81% of the variability in the response could be explained by the model. The “Predicted *R*^2^” of 0.9054 was in reasonable agreement with the “Adjusted *R*^2^” of 0.9774. The “Lack of Fit *F* value” of 1.47 implied that the “Lack of Fit” was not significant relative to the pure error (Table 2). Thus, this model was deemed reliable for analyzing 2,3-BD production.

**Fig. 1:**
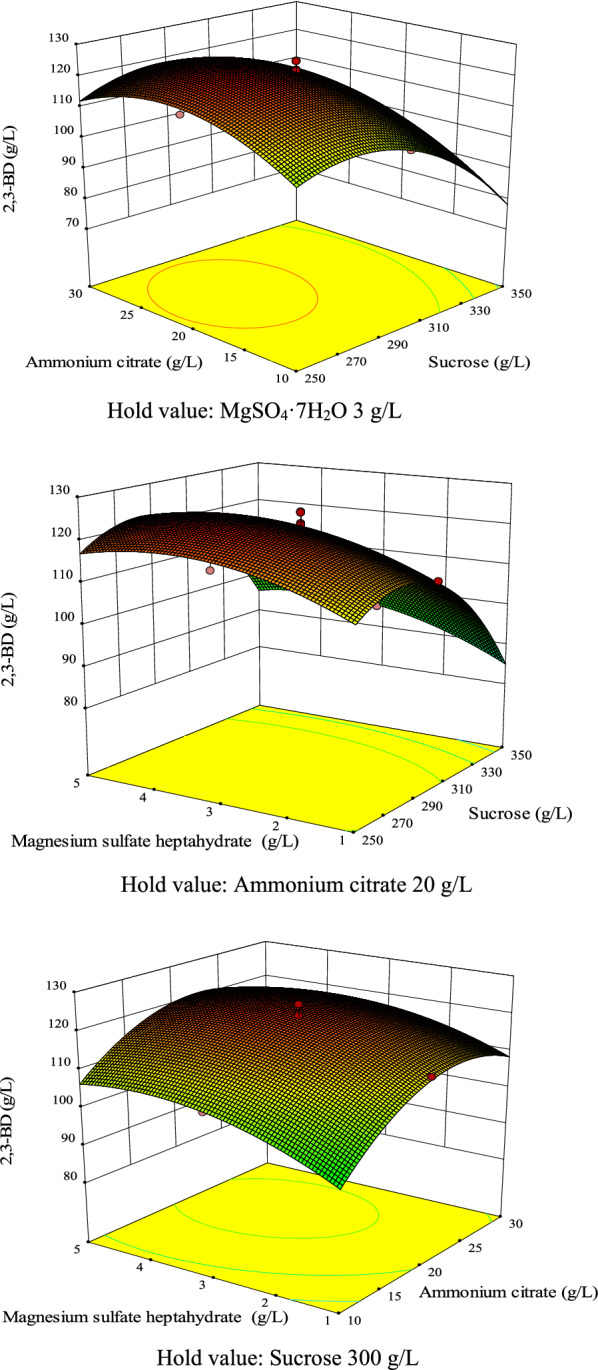
Response surface graphs of 2,3-BD production by *B. subtilis* CS13. **a** Effect of ammonium citrate and sucrose. **b** Effect of MgSO_4_·7H_2_O and sucrose. **c** Effect of MgSO_4_·7H_2_O and ammonium citrate

The effect of these three variables on 2,3-BD production and their optimal levels were further analyzed by RSM. The three-dimensional response surface curves are presented in Fig. 1. The response surface was convex, suggesting the existence of an optimal value for each variable. Each variable value above the optimum value would not be conducive to the production of 2,3-BD. This result was consistent with the negative coefficient estimate of the squared terms. According to the second-order polynomial equation model and response surface analysis, the predicted maximum value of 2,3-BD was 125.0 g/L when the concentrations of sucrose, ammonium citrate, and MgSO_4_·7H_2_O were 281.1, 21.9, and 3.6 g/L, respectively.

RSM was used to find an appropriate medium for 2,3-BD production using *B. subtilis* CS13. As the fermentation time progressed, the maximum value of 2,3-BD in each group of experiments (Table 1) was taken as the response value. However, the reduction in productivity due to substrate inhibition could not be reflected. Therefore, some studies have optimized substrates by setting different sugar concentration gradients before RSM design [17, 20]. In this study, a high concentration of sucrose (250–350 g/L) was used, and significant substrate inhibition occurred at sucrose concentrations above 300 g/L (Table 1).

Ammonium citrate is a significant factor that influences 2,3-BD accumulation in the medium as shown in Table 2 (*P* < 0.0001). *B. subtilis* CS13 production of 2,3-BD requires oxidation of NADH to NAD^+^ [9]. In addition, as a γ-PGA producer, citrate enhances citric acid metabolism, while NH_4_^+^ enhances glutamate metabolism conversion of NADPH to NADP^+^, which subsequently increases NADH accumulation and promotes 2,3-BD synthesis [18, 21]. It is reported that the yield of 2,3-BD can be increased by addition of different organic acid, and ammonium citrate acts as an intermediate metabolite to promote the formation of 2,3-BD [14, 22]. Previous research has also confirmed the significant effects of ammonium citrate for 2,3-BD production in *B. amyloliquefaciens* B10-127 [14]. It is known that α-acetolactate synthase is a key enzyme for 2,3-BD formation, and this enzyme is dependent on Mg^2+^ [23].

### Validation of the optimized medium for 2,3-BD production

**Fig. 2:**
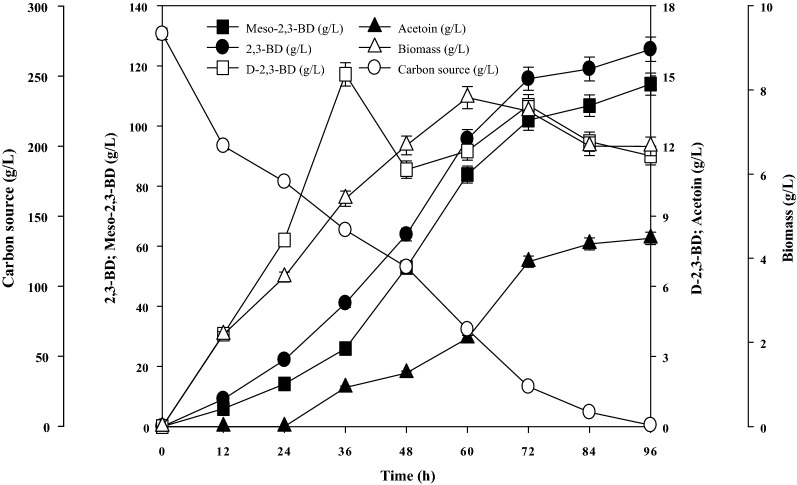
Time course of 2,3-BD production by *B. subtilis* CS13 in the optimized medium

To confirm the reliability of the polynomial equation for predicting 2,3-BD production, a validation experiment was performed in triplicate at the optimal conditions. As shown in Fig. 2, the highest titer of 2,3-BD (125.5 ± 3.1 g/L) was obtained at 96 h, which was very close to the predicted value (120 g/L). Therefore, the optimized medium was good for 2,3-BD production, and the optimal medium composition consisted of 281.1 g/L sucrose, 21.9 g/L ammonium citrate, 1 g/L KH_2_PO_4_, 3.6 g/L MgSO_4_·7H_2_O, 0.04 g/L FeCl_3_·6H_2_O, 0.15 g/L CaCl_2_·2H_2_O, 0.12 g/L MnCl_2_·4H_2_O, and 0.5 g/L NaCl.

### Batch fermentation for 2,3-BD production

**Fig. 3:**
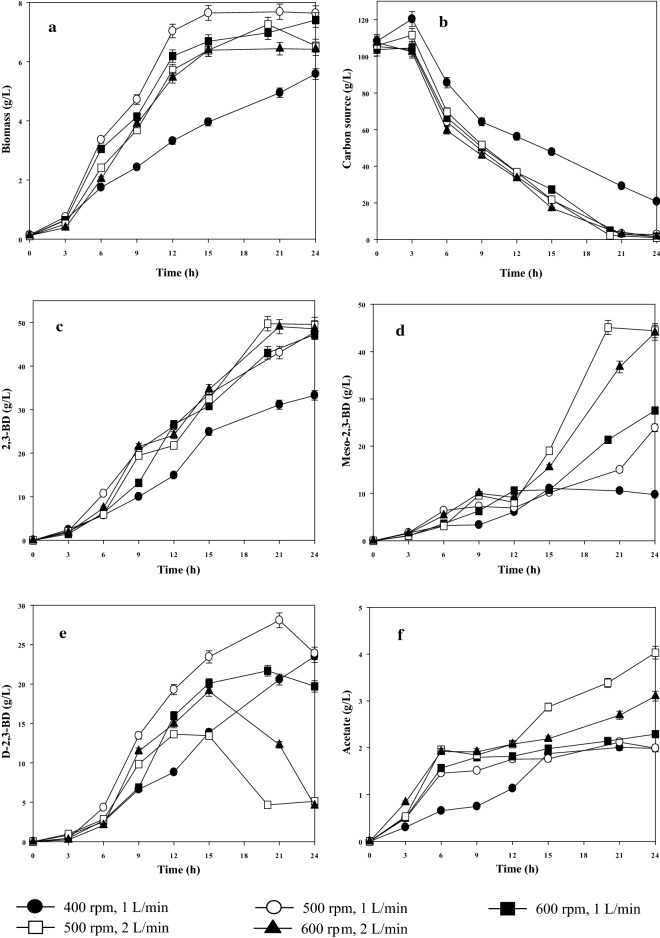
Effects of agitation speed and aeration rate on **a** biomass, **b** sugar consumption, **c** 2,3-BD, **d** meso-2,3-BD, **e** D-2,3-BD, and **f** acetate production in the batch fermentation

The effects of agitation speed (400, 500, and 600 rpm) and aeration rate (1 and 2 L/min) on 2,3-BD production in batch fermentation with the optimal medium at an initial sucrose concentration (100 g/L) were investigated. The highest biomass (7.7 ± 0.3 g/L) was obtained at 500 rpm–1 L/min after 21 h (Fig. 3a). The growth of *B. subtilis* CS13 was low in the agitation speed of 400 rpm, thereby limiting the absorption of the substrate (Fig. 3b). Under other conditions, cell growth and substrate absorption did not show significant differences (Fig. 3a, b). At a high agitation speed (600 rpm) and high aeration (2 L/min), the biomass slightly decreased after 15 h. This result suggests that both a shortage of oxygen and high dissolved oxygen affect the growth of the strain.

The highest titer of 2,3-BD (49.8 ± 1.7 g/L) with the highest productivity (2.49 ± 0.08 g/L/h) was produced under the conditions of 500 rpm–2 L/min. Upon further increasing the agitation speed to 600 rpm at 2 L/min, the final 2,3-BD concentration did not increase (49.0 ± 1.5 g/L) (Fig. 3c). When the aeration was kept at 1 L/min while increasing the agitation speed from 400 to 600 rpm, the titer of 2,3-BD increased from 33.3 ± 1.1 to 47.8 ± 1.5 g/L, and the productivity increased from 1.39 ± 0.05 to 1.99 ± 0.06 g/L/h (Fig. 3c). In addition, meso-2,3-BD increased from 9.8 ± 0.3 to 27.5 ± 0.9 g/L (Fig. 3d). Therefore, the concentration of 2,3-BD can be increased by proper dissolved oxygen, and aeration appears to greatly influence the configuration of 2,3-BD. The titers of meso-2,3-BD (45.01 ± 1.3 and 44.0 ± 1.1 g/L) were obtained under the aeration rate of 2 L/min at 500 and 600 rpm conditions, respectively, and meso-2,3-BD accounted for 90% of the total 2,3-BD (Fig. 3d). D-2,3-BD is more likely to be synthesized under a low aeration rate (1 L/min) (Fig. 3e). These results indicate that high concentration and high productivity of 2,3-BD can be achieved simultaneously at a speed of 500 rpm and aeration rate of 2 L/min.

It is known that agitation speed and aeration rate affect dissolved oxygen during the fermentation process. One study found that low oxygen is advantageous for 2,3-BD production using *B. subtilis* but hampers cell growth and substrate absorption, whereas high dissolved oxygen promotes the production of acetoin [9]. However, in this work, the 2,3-BD yield increased from 0.38 to 0.43 g_2,3-BD_/g_sucrose_ as the agitation speed increased from 400 to 600 rpm at an aeration rate of 1 L/min. The highest yield of 0.48 g_2,3-BD_/g_sucrose_ was obtained under the conditions of 500 rpm–2 L/min. Therefore, a suitable oxygen supply is beneficial to 2,3-BD production by *B. subtilis* CS13. Interestingly, acetoin was not detected during the batch fermentation process. During the *B. subtilis* growth phases, high consumption rates of NADH and NAD^+^ were observed, resulting in an NADH/NAD^+^ ratio that was lower intracellularly; thus, acetoin to the 2,3-BD pathway is enhanced to regenerate the excess reducing power [6]. Zhang et al. [24] reported that a high yield of acetoin was formed at the decline phase of fermentation. Oxygen supply affects the configuration of 2,3-BD, and low dissolved oxygen is beneficial for D-2,3-BD synthesis [25]. In the current work, the lower the dissolved oxygen value, the more D-2,3-BD was formed (Fig. 3e). At the aeration rate of 1 L/min, the concentration of D-2,3-BD slightly decreased at 400 rpm compared to 500 rpm, likely due to the shortage of oxygen that limited the consumption of sugars. During fermentation with the higher agitation speeds (500 and 600 rpm) and aeration rate (2 L/min), the purity of meso-2,3-BD increased to 90.5%, suggesting that a moderate increase in oxygen supply may also increase the purity of meso-2,3-BD produced using *B. subtilis* CS13. More interestingly, at higher dissolved oxygen conditions, the D-2,3-BD concentration displayed a decrease after a certain value. This phenomenon is the first of its kind to be reported and may have been caused by the NADH/NAD^+^ ratio in the cells [25].

The earlier studies found that supplementation with acetate enhanced the 2,3-BD production [26, 27]. Acetate can induce α-acetolactate synthase (α-ALS), α-acetolactate decarboxylase (α-ALD), diaceyl (acetoin) reductase (DAR), and butanediol dehydrogenase (BDH), which enhanced the pathway of 2,3-BD and reduces the acetoin to 2,3-BD. Also, acetate acts as an inhibitor for the oxidation of 2,3-BD to acetoin [28]. As shown in Fig. 3f, the highest titer of 4.0 ± 0.1 g/L acetate was obtained at 500 rpm–2 L/min. Although the agitation speed of 500 rpm was higher than previous reports [9, 10, 13], the yield of 2,3-BD did not decrease. The production of 2,3-BD at a relatively high agitation speed is the first report, the increase in the yield of 2,3-BD might be due to the formation of acetate. Future studies will be conducted in the gene and metabolic regulation.

### Fed-batch production of 2,3-BD from sucrose

**Fig. 4:**
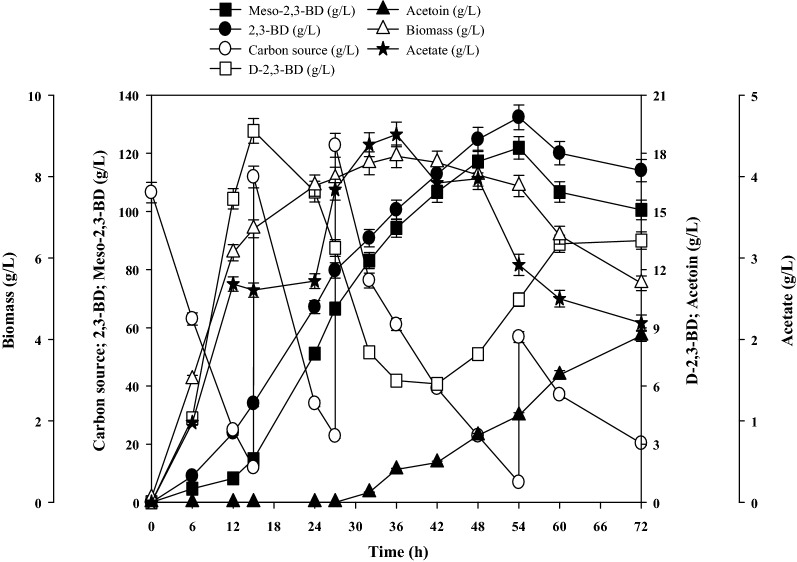
Time course of fed-batch fermentation of 2,3-BD using sucrose as the carbon source by *B. subtilis* CS13

To eliminate the effect of substrate inhibition, fed-batch cultivation with a low initial sucrose concentration (100 g/L) in the optimized medium was further studied to increase 2,3-BD productivity and support the effectiveness of industrial production. From Fig. 4, we concluded that the biomass increased to 8.5 ± 0.3 g/L at 36 h and then slowly decreased. 2,3-BD production reached a maximum value of 132.4 ± 4.4 g/L at 54 h with a productivity of 2.45 ± 0.08 g/L/h and yield of 0.45 g_2,3-BD_/g_sucrose_. The highest titer of meso-2,3-BD reached 121.9 ± 3.8 g/L, whereas the concentration of D-2,3-BD changed considerably during fermentation until finally stabilizing at 13.5 ± 0.7 g/L.

In this study, fed-batch fermentation for the production of 2,3-BD by various bacterial species was demonstrated (Table 3). The highest 2,3-BD titer was achieved by *K. pneumoniae* SDM with the highest productivity of 4.21 g/L/h [29]. However, *K. pneumoniae* is a pathogenic microorganism, which poses a risk in industrial applications. When BDH and glyceraldehyde-3-phosphate dehydrogenase were co-overproduced in *B. amyloliquefaciens*, the 2,3-BD concentration improved from 112.3 to 132.9 g/L [6]. *B. licheniformis* DSM 8785 produced 144.7 g/L 2,3-BD over 127 h of fed-batch fermentation [30] with a productivity of only 1.14 g/L/h. Other strains, such as *K. oxytoca* GSC 12206 and *E. aerogenes* KCTC 2190, displayed high productivity but relatively low concentrations of 2,3-BD [31, 32]. For comparison, the maximum 2,3-BD concentration obtained from *B. subtilis* CS13 was 132.4 g/L with a productivity of 2.45 g/L/h. To our knowledge, this is the highest 2,3-BD production level reported for *B. subtilis*. A high 2,3-BD titer makes downstream processing much easier, and the high productivity of *B. subtilis* CS13 reduces the fermentation time. Therefore, we consider *B. subtilis* CS13 to be a very promising strain for large-scale production of 2,3-BD in industrial contexts.

**Table 3:** Production of 2,3-BD by various microorganisms

Strain	Substrate	Operation mode	2,3-BD titer (g/L)	2,3-BD productivity (g/L/h)	2,3-BD yield (g _2,3-BD_/g _substrate_)	References
*B. subtilis* 168 (BSF9)	Glucose	Fed-batch	103.7	0.459	0.487	[9]
*B. subtilis* TUL322	Glucose	Fed-batch	75.73	0.66	0.31	[42]
*B. amyloliquefaciens* PBG	Glucose	Fed-batch	132.9	2.95	0.43	[6]
*B. licheniformis* DSM 8785	Glucose	Fed-batch	144.7	1.14	0.42	[30]
*B. vallismortis* B-14891	Glucose	Batch	60.4	1.10	0.33	[44]
*S. cerevisiae* CEN.PK2-1C	Glucose	Fed-batch	72.9	1.43	0.41	[45]
*S. cerevisiae* YHI030	Glucose	Fed-batch	81	0.26	0.41	[46]
*K. oxytoca* ME-UD-3	Glucose	Fed-batch	130	1.63	0.48	[47]
*K. oxytoca* GSC 12206	Glucose	Fed-batch	115	2.27	0.41	[31]
*K. pneumoniae* KG1	Glucose	Fed-batch	116	2.23	0.49	[5]
*K. pneumoniae* SDM	Glucose	Fed-batch	150	4.21	0.43	[29]
*E. aerogenes* KCTC 2190	Glucose	Fed-batch	118	2.18	0.42	[32]
*P. polymyxa* CJX518	Glucose	Fed-batch	71.7	1.33	0.39	[25]
*B. subtilis* CS13	Sucrose	Fed-batch	132.4	2.45	0.45	This work

In the fed-batch fermentation process of *B. subtilis* CS13, acetoin as the main by-product appeared after 30 h and continued to increase over time. After 54 h, the 2,3-BD concentration decreased, while the acetoin concentration increased from 4.4 ± 0.1 to 8.6 ± 0.2 g/L, indicating that the ratio of NADH/NAD^+^ had changed, and 2,3-BD was converted to acetoin in the final phase of fermentation [13]. On the other hand, the concentration of acetate increased to 4.5 ± 0.2 g/L at 36 h and then decreased to 2.2 ± 0.1 g/L by the end of fermentation (Fig. 4). The reduced inhibition of acetate might be promoted the production of acetoin. The concentration of 2,3-BD reached its highest level, and the ratio of meso-2,3-BD/2,3-BD was 92.1% at 54 h. The stereoisomers of 2,3-BD are microorganism dependent. *P. polymyxa* produced D-2,3-BD as the major product [33], while *K. pneumonia* and *B. subtilis* produced meso-2,3-BD as the major product [3, 10] and *B. licheniformis* produced a mixture of D-2,3-BD and meso-2,3-BD at a ratio of nearly 1:1 [17]. Thus, the key enzymes for 2,3-BD isomer formation are different among the various 2,3-BD producers. In *B. subtilis* CS13, meso-2,3-butanediol dehydrogenase (meso-BDH) appears to show greater activity toward meso-2,3-BD than D-2,3-butanediol dehydrogenase (D-BDH) does toward D-2,3-BD formation. Meso-2,3-BD is an important platform chemical with numerous special applications, such as used for microbial production of 2-butanol and butanone [34, 35], as well as producing renewable polyesters and enantiomerically pure halohydrins [36, 37]. The high ratio of meso-2,3-BD/2,3-BD will simplify the purification process and improve commercial viability.

### Fed-batch production of 2,3-BD from untreated molasses

**Fig. 5:**
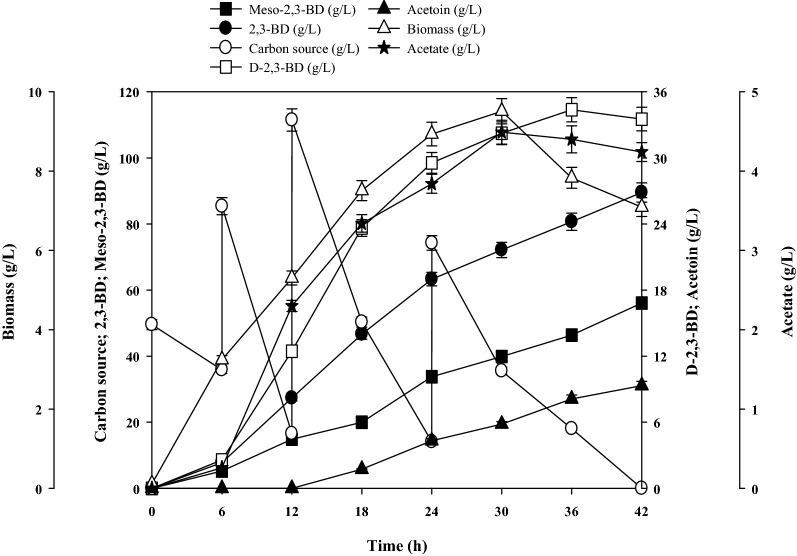
Time course of fed-batch fermentation of 2,3-BD using untreated molasses as the carbon source by *B. subtilis* CS13

Finally, we tested the feasibility of using untreated molasses as a low-cost carbon source in the fed-batch production of 2,3-BD by *B. subtilis* CS13. Under the conditions of 500 rpm–2 L/min, all of the sugar was consumed at 42 h, and 89.6 ± 2.8 g/L 2,3-BD was obtained with a productivity of 2.13 ± 0.07 g/L/h (Fig. 5). After fermentation, the broth volume increased due to the addition of a large amount of untreated molasses, which had the potential to decrease the concentration of 2,3-BD. In reality, a total of 693.6 ± 10.5 g untreated molasses containing 332.8 ± 5.2 g sugar was consumed, producing 140.2 ± 3.1 g 2,3-BD at a yield of 0.42 g_2,3-BD_/g_sugar_ after 42 h. The increase in the volume of the fermentation broth also reduced the dissolved oxygen, and the final meso-2,3-BD and D-2,3-BD titers were 56.1 ± 1.7 and 33.5 ± 1.1 g/L, respectively. The low dissolved oxygen was of significance to acetoin accumulation [38], and the yield of acetoin increased to 9.3 ± 0.3 g/L after 42 h with acetate yield of 4.4 ± 0.2 g/L. Furthermore, the maximum biomass of 9.5 ± 0.4 g/L was obtained at 30 h (Fig. 5), which proved to be better than using sucrose (8.5 ± 0.3 g/L) as the carbon source. Some reports have suggested that untreated molasses contains some unknown essential nutrients, such as crude protein and vitamins, that may stimulate cell growth [39]. After 30 h, the biomass began to decrease gradually, potentially caused by the high concentration of molasses containing metal ions and ash that exceeded the strain tolerance limit, which could be toxic for cells [40]. Related to this, the 2,3-BD productivity decreased to 1.45 ± 0.04 g/L/h compared to the first 30 h of 2.40 ± 0.07 g/L/h.

Using molasses as a substrate to produce 2,3-BD has been previously reported [30, 31]. *E. cloacae* produced a high concentration of 2,3-BD (90.8 g/L) with a productivity of 1.51 g/L/h and yield of 0.39 g_2,3-BD_/g_molasses_ [41]. *B. subtilis* TUL322 produced 75.73 g/L 2,3-BD with a productivity of 0.66 g/L/h by feeding glucose into the molasses-based medium [42]. Interestingly, lower concentrations of 2,3-BD (78.9 g/L and 76.2 g/L) were produced by *K. pneumonia* SDM and *K. pneumonia* ATCC200721 by feeding corncob molasses and sugarcane molasses, respectively [16, 43]. Compared to previous research, *B. subtilis* CS13 appears to utilize untreated molasses more efficiently for the production of 2,3-BD, demonstrating a higher titer, productivity, and yield in the present study.

## Conclusions

In this study, a high titer of 2,3-BD from sucrose was obtained by *B. subtilis* CS13 using optimized medium. The concentration and configuration of 2,3-BD as well as the synthesis of acetoin were significantly affected by the agitation speed and aeration rate in the batch fermentation process. In fed-batch fermentation, a maximum 2,3-BD concentration of 132.4 ± 4.4 g/L with a productivity of 2.45 ± 0.08 g/L/h and yield of 0.45 g_2,3-BD_/g_sucrose_ was obtained. This strain produced meso-2,3-BD and D-2,3-BD at a ratio of 92.1:7.9. Furthermore, a high concentration, productivity, and yield of 2,3-BD were able to be achieved using untreated molasses as the carbon source. Therefore, *B. subtilis* CS13 constitutes a promising 2,3-BD producer using a low-cost medium on an industrial scale.

## Data Availability

All data generated or analyzed during this study are included in this article.

## Abbreviations

2,3-BD: 2,3-Butanediol
*B. subtilis*: *Bacillus subtilis*
RSM: Response surface methodology
HPLC: High-performance liquid chromatography
FCCD: Face-centered central composite design
BDH: 2,3-Butanediol dehydrogenase
D-BDH: D-2,3-butanediol dehydrogenase
meso-BDH: Meso-2,3-butanediol dehydrogenase
